# Two-Stage Conservative Management of Mandibular Unicystic Ameloblastoma with an Intraluminal Pattern: A Case Report

**DOI:** 10.3390/dj14080524

**Published:** 2026-08-17

**Authors:** Andrea Chango-Chávez, Marco Vizuete-Bolaños, Stephany Paladines-Calle, Juan Marcos Parise-Vasco

**Affiliations:** 1 Posgrado de Cirugía Oral, Universidad Central del Ecuador, Quito 170521, Ecuador; avchango@uce.edu.ec; 2 Cirugía Maxilofacial, Hospital General Docente de Calderón, Quito 170208, Ecuador; marcco.vizuete@hgdc.gob.ec; 3 Carrera de Odontología, Universidad Politécnica Salesiana, Cuenca 010101, Ecuador; spaladines@ups.edu.ec; 4 Center for Evidence Ecosystems, Implementation Science, and Decision-Making (CIDES), Facultad de Ciencias de la Salud y Bienestar Humano, Universidad Tecnológica Indoamérica, Ambato 180150, Ecuador

**Keywords:** ameloblastoma, oral surgical procedures, conservative treatment, healthcare, quality of life, case reports

## Abstract

Intraluminal unicystic ameloblastoma is characterized by tumor proliferation confined to the cystic lumen, without invasion of the supporting connective tissue, a feature that has led some authors to regard it as particularly suited to conservative surgical treatment. **Objective:** This study aims to describe the conservative two-stage management of a mandibular unicystic ameloblastoma with an intraluminal pattern, treated by decompression followed by enucleation with the aim of avoiding mandibular resection. **Case Report:** A 24-year-old female patient presented with a slight swelling in the right mandibular angle that had evolved over two years without prior treatment. Panoramic radiography revealed a well-defined radiolucent lesion extending from the mesial surface of the right mandibular second molar through the mandibular ramus to the ipsilateral coronoid process. Cone-beam computed tomography (CBCT) confirmed the lesion as a hypodense area measuring approximately 6 × 3.4 × 2.9 cm. A two-stage surgical approach was performed: the first stage consisted of an incisional biopsy and the placement of a decompression tube for six months; the second stage consisted of enucleation of the lesion. The definitive histopathological diagnosis was intraluminal unicystic ameloblastoma with a plexiform growth pattern. The surgical procedures were completed without preoperative, intraoperative, or postoperative complications. Follow-up evaluations were performed at 3 and 6 months and at 1 and 2 years after the initial surgical stage. **Conclusions:** At 24 months from the initiation of treatment, the outcome was favorable, with evidence of mandibular bone apposition and no signs of lesion recurrence.

## 1. Introduction

According to the 2022 World Health Organization classification [1], four variants are recognized: conventional ameloblastoma, formerly referred to as solid/multicystic; unicystic ameloblastoma; peripheral or extraosseous ameloblastoma; and metastasizing ameloblastoma [2]. Ameloblastoma represents approximately 1% of all head and neck neoplasms [1] and 11% of odontogenic tumors [2].

Whereas conventional ameloblastoma typically presents in the fourth to fifth decades of life and exhibits more aggressive behavior, unicystic ameloblastoma occurs more frequently in the second or third decade and follows a less aggressive biological course [1,3]. According to Ackermann’s histological classification, three subtypes are recognized—luminal (without epithelial infiltration into the lumen or the tumor capsule), intraluminal (without infiltration of the connective tissue), and mural (invasion of the epithelium into the tumor capsule)—which differ in their invasive potential and, consequently, in their prognosis and therapeutic implications [4,5].

Surgical management depends on the histological subtype, size, location, and extent of the lesion, as well as on the patient’s age and systemic condition [6]. Surgery remains the treatment of choice and may involve either conservative techniques—such as marsupialization, decompression, and enucleation with curettage—or radical procedures, including marginal or segmental resection [6]. Reported recurrence rates range from 15% to 25% after radical treatment and from 55% to 90% after conservative approaches [7].

Although marsupialization and decompression of unicystic ameloblastomas share the same objective—reducing intraluminal pressure and, consequently, the size of the lesion—they differ in technique. Marsupialization sutures the fibrous cyst wall to the adjacent oral mucosa, creating permanent communication between the cystic lumen and the oral cavity, whereas decompression maintains this communication by means of a decompression device [8].

Conservative management has been shown to provide favorable results in the luminal and intraluminal variants of unicystic ameloblastoma, particularly in young patients [5], and in cases without imaging evidence of root resorption or cortical perforation [9]. However, this approach carries a higher risk of local recurrence; therefore, segmental resection with safety margins remains the standard treatment for most ameloblastomas [10]. The conservative approach helps preserve the pulp vitality of the adjacent teeth, the inferior alveolar nerve, and the contour of the mandibular bone [11]. In contrast, mandibular resection is associated with more severe complications, including facial deformity, pathological fracture, tooth loss, and neurosensory disturbances, often requiring complex reconstructive procedures for functional rehabilitation [7].

Controversy remains regarding which histological subtypes and lesion dimensions are optimal candidates for conservative management, as well as the recurrence rates expected with this approach. In particular, evidence remains limited for the intraluminal subtype—considered the least aggressive variant [9]—when treated by decompression followed by enucleation. From this perspective, the present case contributes to the limited body of evidence on conservative management of the intraluminal subtype. It underscores the value of an integrated clinical, radiographic, and histopathological correlation in patient selection and illustrates a staged protocol that aims to reduce tumor size before definitive surgery, thus favoring anatomical and functional preservation.

In this context, the objective of this case report is to describe the conservative two-stage management of a unicystic mandibular ameloblastoma with an intraluminal pattern through decompression followed by enucleation, with the aim of avoiding mandibular resection and documenting clinical and imaging results after two years of follow-up from the initiation of treatment. This case report was prepared according to the Surgical Case Report (SCARE) guidelines [12].

## 2. Case Report

**Figure 1:**
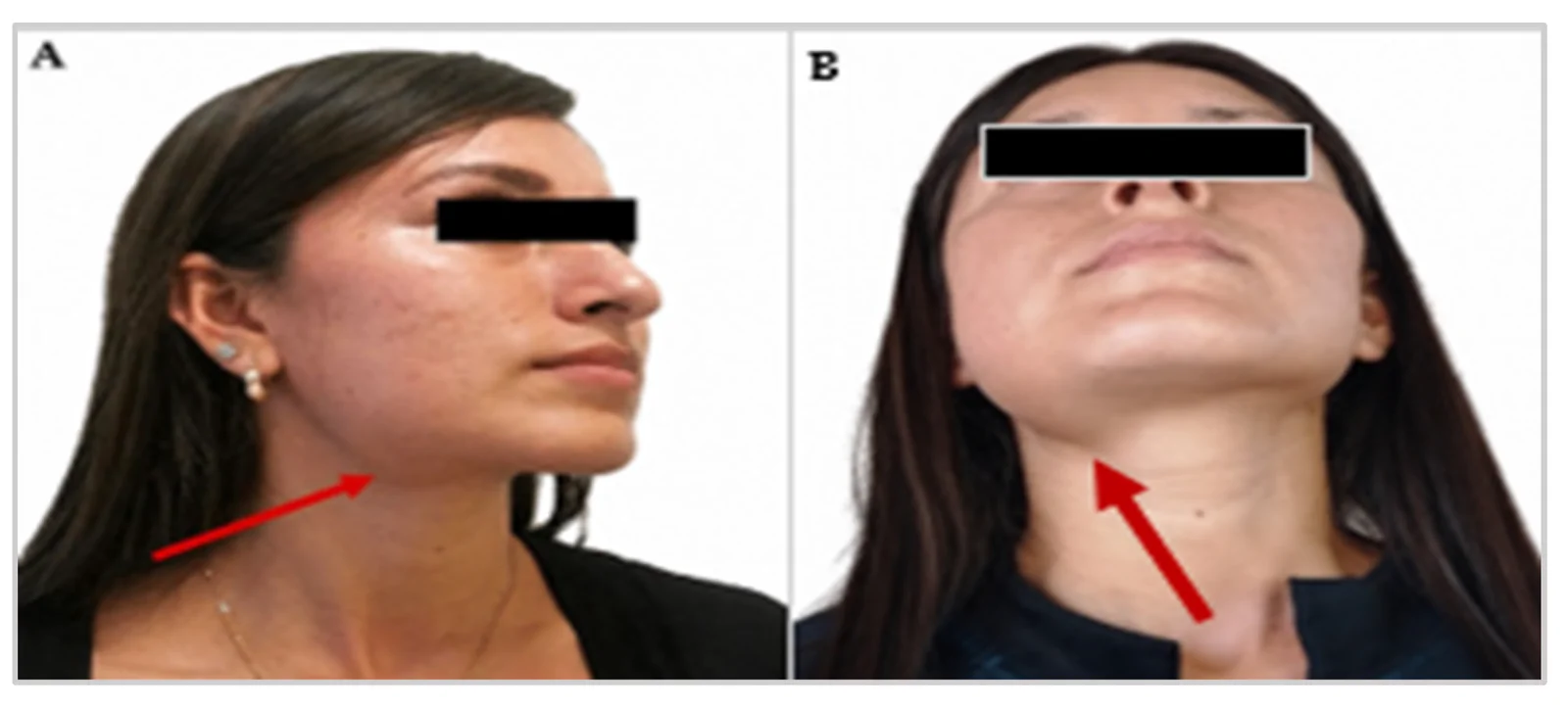
Preoperative extraoral clinical photographs. (**A**) Lateral view. (**B**) Submental view. The red arrows indicate the mild facial asymmetry in the right submandibular region.

A 24-year-old mestizo female patient, with no relevant medical, surgical or pharmacological history, presented to the Department of Maxillofacial Surgery with a slight swelling in the right mandibular angle region that had been present for approximately two years, without prior treatment. She reported no family history of odontogenic or maxillofacial disease. Extraoral clinical examination revealed mild facial asymmetry in the right submandibular region (Figure 1), without erythema or increased local temperature. Intraoral examination showed normal-appearing mucosa, without palpable buccal or lingual cortical expansion.

**Figure 2:**
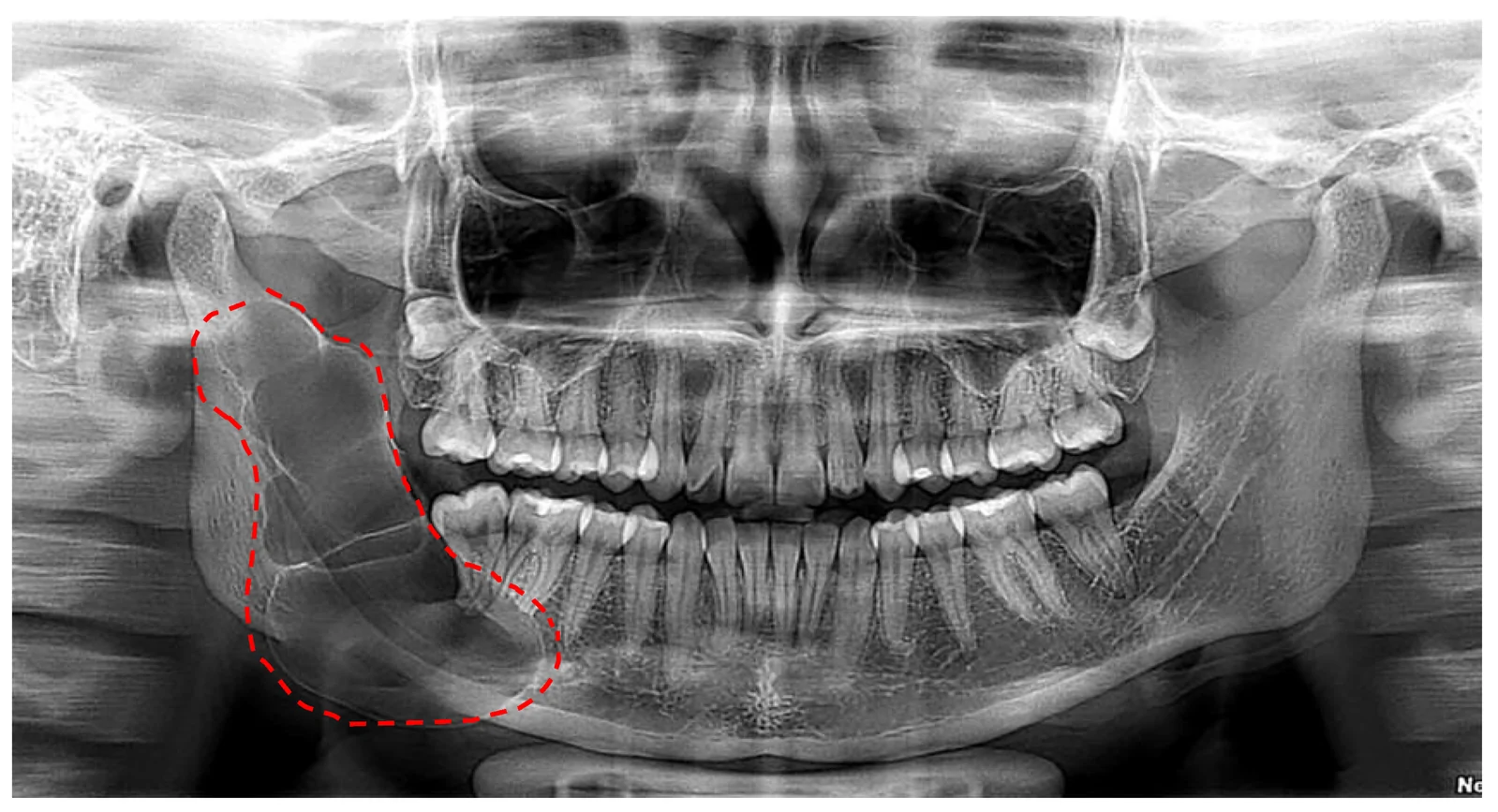
Initial panoramic radiograph. The dashed red outline delineates a unilocular radiolucent lesion with well-defined corticated margins extending from the mesial surface of the right mandibular second molar through the mandibular ramus to the ipsilateral coronoid process.

**Figure 3:**
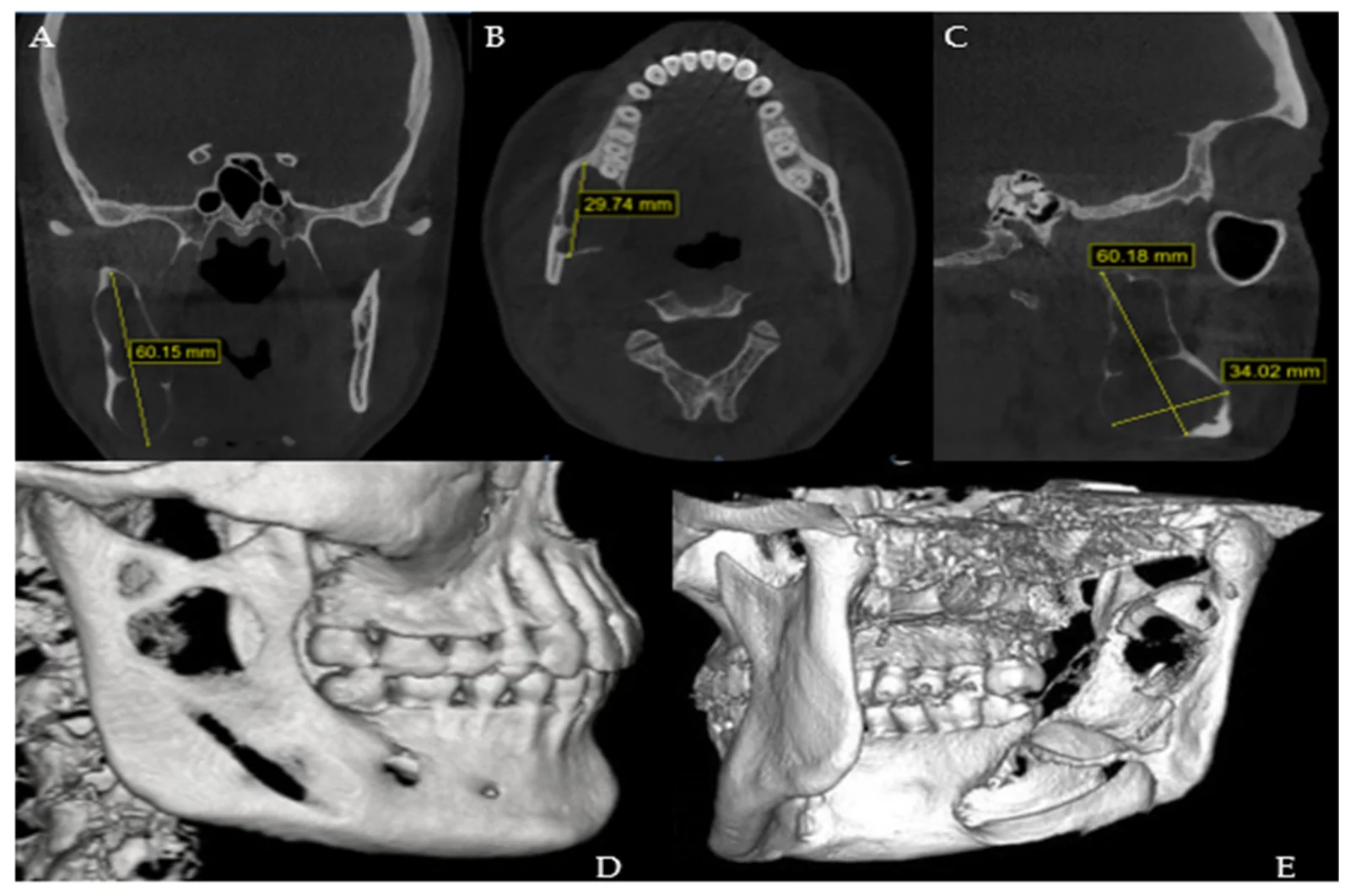
Initial cone-beam computed tomography (CBCT). (**A**) Coronal, (**B**) axial, and (**C**) sagittal sections; (**D**) right lateral and (**E**) left posterior oblique views of the mandibular tomographic reconstruction. The lesion appears as a well-defined hypodense area without cortical perforation or root resorption. Yellow measurement lines indicate the initial lesion dimensions (approximately 6 × 3.4 × 2.9 cm).

On imaging examination, panoramic radiography revealed a unilocular radiolucent lesion with well-defined corticated margins that extended from the mesial surface of the right mandibular second molar through the mandibular ramus to the ipsilateral coronoid process (Figure 2). Cone-beam computed tomography (CBCT) confirmed the lesion as a hypodense area in the same location, measuring approximately 6 × 3.4 × 2.9 cm, without evidence of cortical perforation, root resorption, or involvement of the inferior alveolar nerve (Figure 3). Based on these imaging characteristics, the differential diagnosis considered included conventional (solid/multicystic) ameloblastoma, dentigerous cyst, and odontogenic keratocyst. Given the well-defined unilocular appearance, the non-aggressive radiographic behavior, and the clinical findings, a presumptive diagnosis of unicystic ameloblastoma was established.

After multidisciplinary evaluation, a two-stage conservative treatment plan was chosen. The first stage consisted of an incisional biopsy to confirm the presumptive histopathological diagnosis, together with the placement of a decompression tube intended to reduce the size of the lesion prior to definitive enucleation; the second stage consisted of enucleation of the lesion.

**Figure 4:**
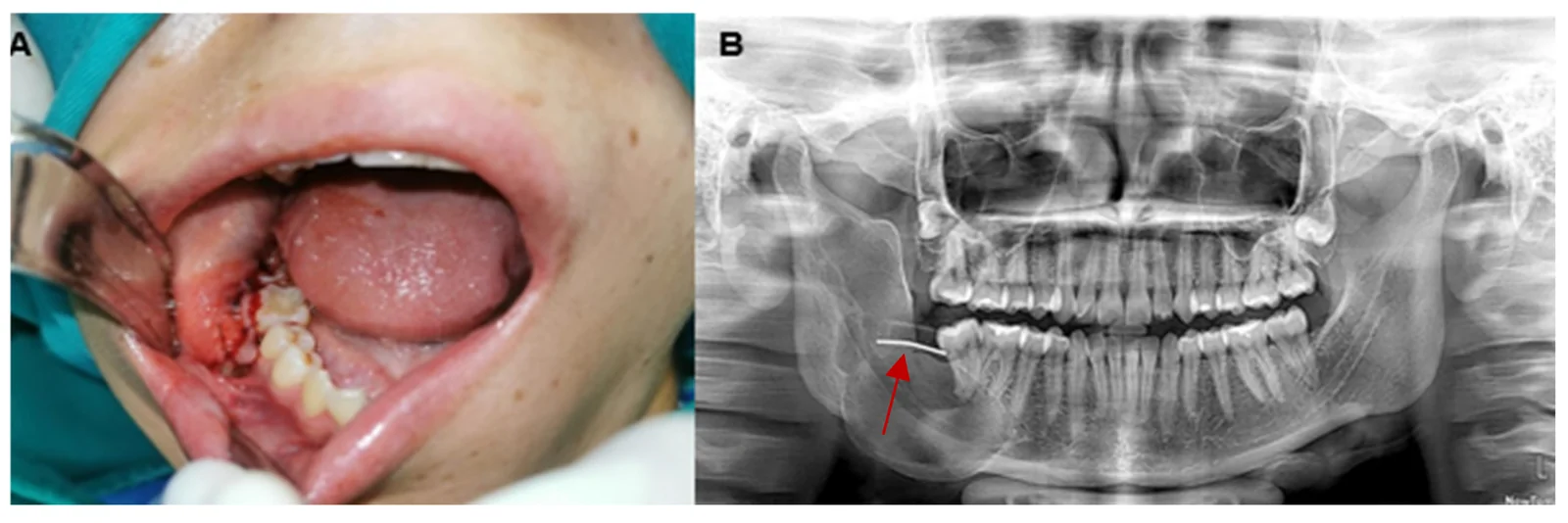
Placement of the decompression tube. (**A**) Intraoperative clinical photograph showing the tube secured within the surgical site. (**B**) Panoramic radiograph; the red arrow indicates the radiolucent shadow of the decompression tube at the site of the lesion.

In the first surgical stage, performed under local infiltrative anesthesia with 2% lidocaine and 1:100,000 epinephrine, a full-thickness mucoperiosteal flap was elevated, and an osteotomy of the buccal cortex was performed to access the cystic cavity. A specimen was obtained by incisional biopsy and sent for histopathological examination. During the same procedure, a decompression tube (a segment of a dental saliva ejector) was inserted through the bony window and secured to the mucosa with 4-0 nylon suture to fix the device; the cyst wall was not marsupialized to the oral mucosa (Figure 4). The patient was discharged on the same day with postoperative instructions. The tube was maintained for 6 months, during which the patient was instructed to perform irrigation three times daily with 0.9% sodium chloride solution (physiological saline) through the decompression tube to maintain patency and prevent local infection. Postoperative pharmacological management for this stage included amoxicillin 500 mg every 8 h for 7 days, paracetamol 1 g every 8 h for 5 days, and ibuprofen 400 mg every 8 h for 3 days. During the six-month decompression phase, the patient was reviewed on an outpatient basis at 8 days and at 1, 3, and 6 months postoperatively; throughout this period the decompression tube remained patent and in adequate position, and the clinical course was uneventful, with no signs of local infection and no neurosensory disturbance.

Histopathological examination of the incisional biopsy confirmed the diagnosis of unicystic ameloblastoma. The second surgical stage was performed six months after the initiation of decompression: the decompression tube was removed and the lesion was enucleated under general anesthesia. The enucleated specimen consisted of multiple irregular soft-tissue fragments, light-brown in color and soft in consistency, together measuring approximately 4.0 × 3.0 × 0.9 cm, which were sent for histopathological analysis.

**Figure 5:**
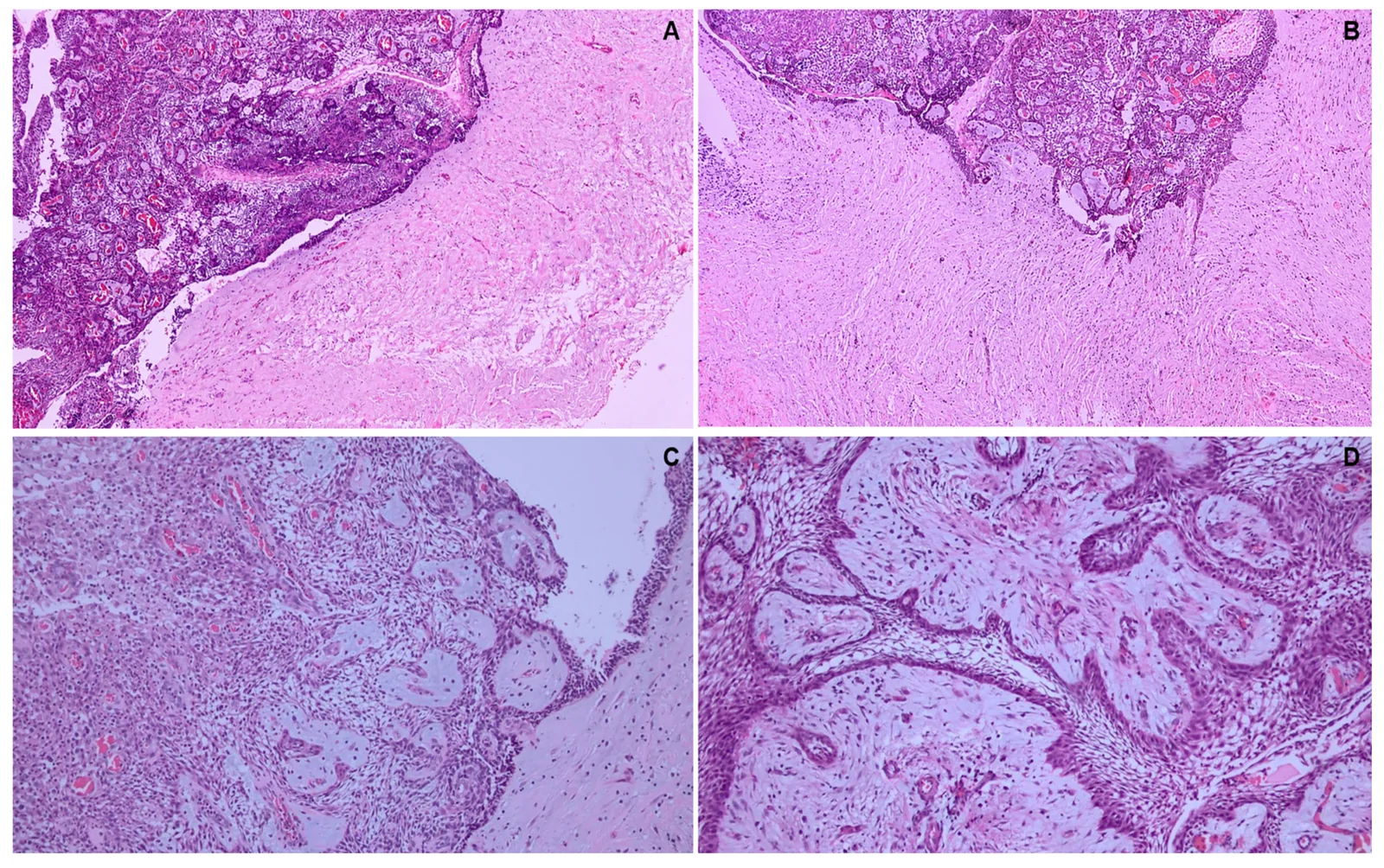
Photomicrographs of the enucleated specimen (hematoxylin and eosin). (**A**,**B**) Low-power views (×4 objective; ×40 total magnification) showing the interface between the ameloblastomatous proliferation and the cyst wall: the tumor epithelium is confined to the luminal aspect and remains in continuity with the cystic lining, whereas the subjacent fibrous connective tissue capsule is devoid of epithelial islands or strands. (**C**) ×20 objective (×200 total magnification), showing plexiform ameloblastomatous epithelium admixed with chronic inflammatory cells. (**D**) ×40 objective (×400 total magnification), showing anastomosing epithelial cords with a peripheral layer of palisaded hyperchromatic ameloblastic cells.

Histopathological analysis was performed on haematoxylin and eosin (H&E)-stained sections; no immunohistochemical study was performed and the diagnosis was established on histomorphological grounds. The specimen was embedded in its entirety, yielding three paraffin blocks from which one H&E-stained section per block was examined (three sections in total). Microscopic examination revealed a cystic lesion lined by ameloblastic epithelium, with a plexiform epithelial proliferation projecting into the cystic lumen; the peripheral cells showed ameloblastic morphology with palisaded hyperchromatic nuclei, and the central areas were loosely arranged, resembling stellate reticulum. These findings were admixed with a severe, diffuse chronic inflammatory infiltrate, consistent with the preceding decompression treatment. Sampling encompassed the thickness of the cyst wall as represented in the material received, and the fibrous connective tissue capsule examined was free of islands or strands of ameloblastomatous epithelium. Areas of haemorrhage and mature lamellar bone were also present. Based on these criteria, the lesion was classified as an intraluminal unicystic ameloblastoma according to Ackermann’s classification, with a plexiform growth pattern (Figure 5).

The immediate postoperative course was uneventful. The patient remained hospitalized for 48 h and received 1.5 g of intravenous ampicillin/sulbactam every 8 h as antibiotic therapy and 1 g of intravenous paracetamol for analgesia. The sutures were removed on postoperative day 8. Intraoral examination showed adequate healing of the surgical wound, with no signs of infection, inflammation, or dehiscence.

**Figure 6:**
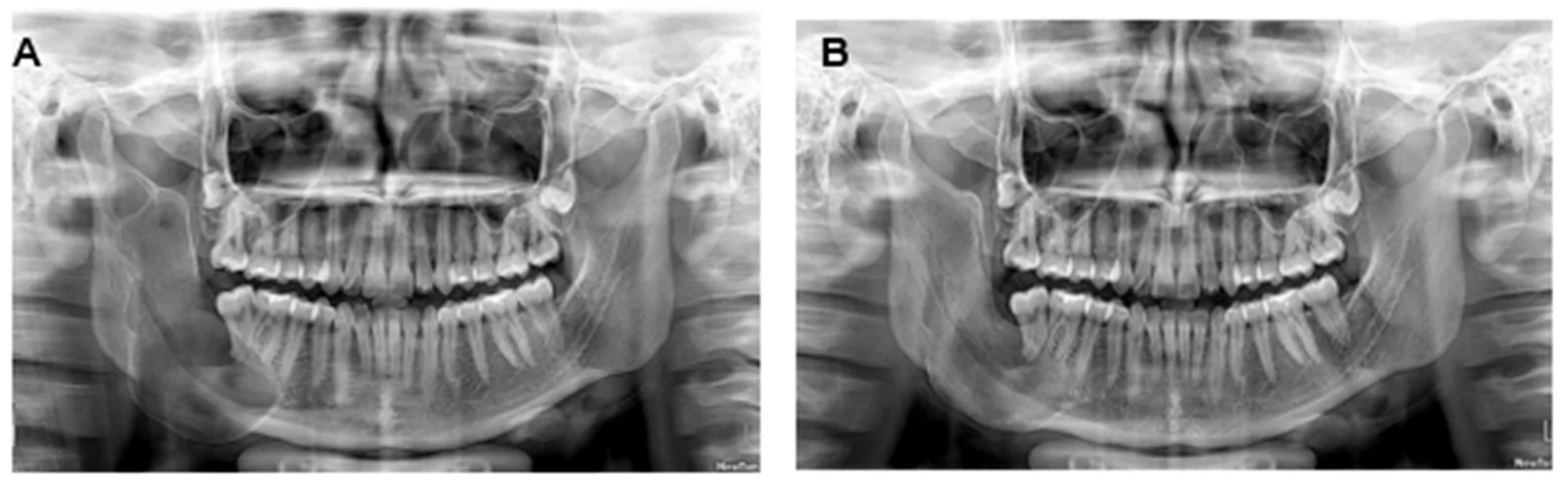
Postoperative panoramic radiographs after enucleation. (**A**) At 3 months and (**B**) 6 months after the second surgical stage, showing progressive bone healing of the surgical site without evidence of residual lesion or recurrence.

**Figure 7:**
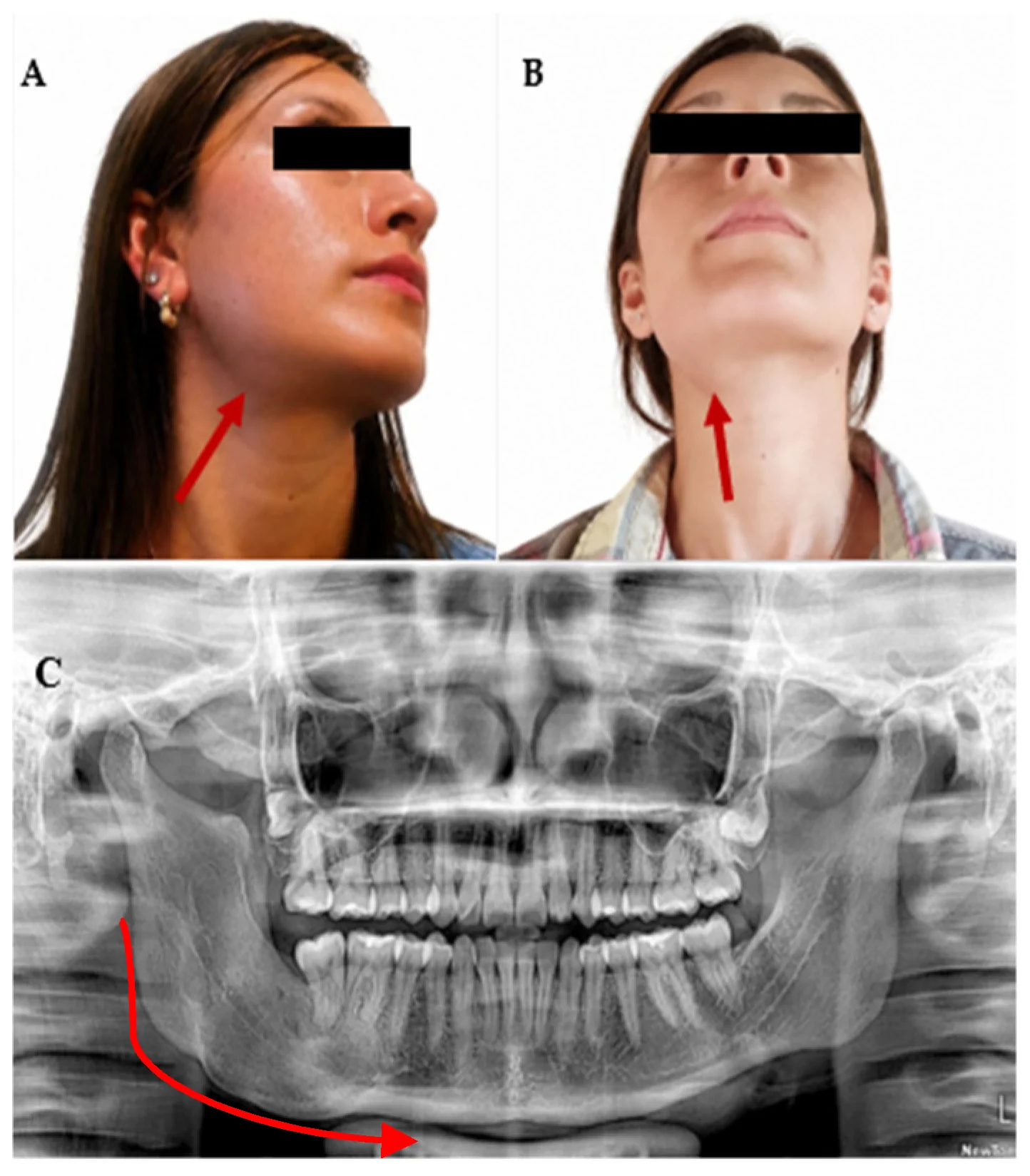
Clinical and radiographic follow-up at 1 year. (**A**) Lateral extraoral view. (**B**) Submental extraoral view. (**C**) Panoramic radiograph at 1-year follow-up. The red arrows indicate improved facial symmetry in the right submandibular region and restoration of anatomical contour.

Clinical and radiographic follow-up was performed. Postoperative panoramic radiographs at 3 and 6 months after enucleation showed progressive bone healing of the surgical site without evidence of residual lesion or recurrence (Figure 6). Proportional bone apposition was observed, and complete new bone formation was documented in the mandible at 1 year of follow-up after enucleation, with restoration of the anatomical contour and consequent recovery of facial symmetry (Figure 7).

**Figure 8:**
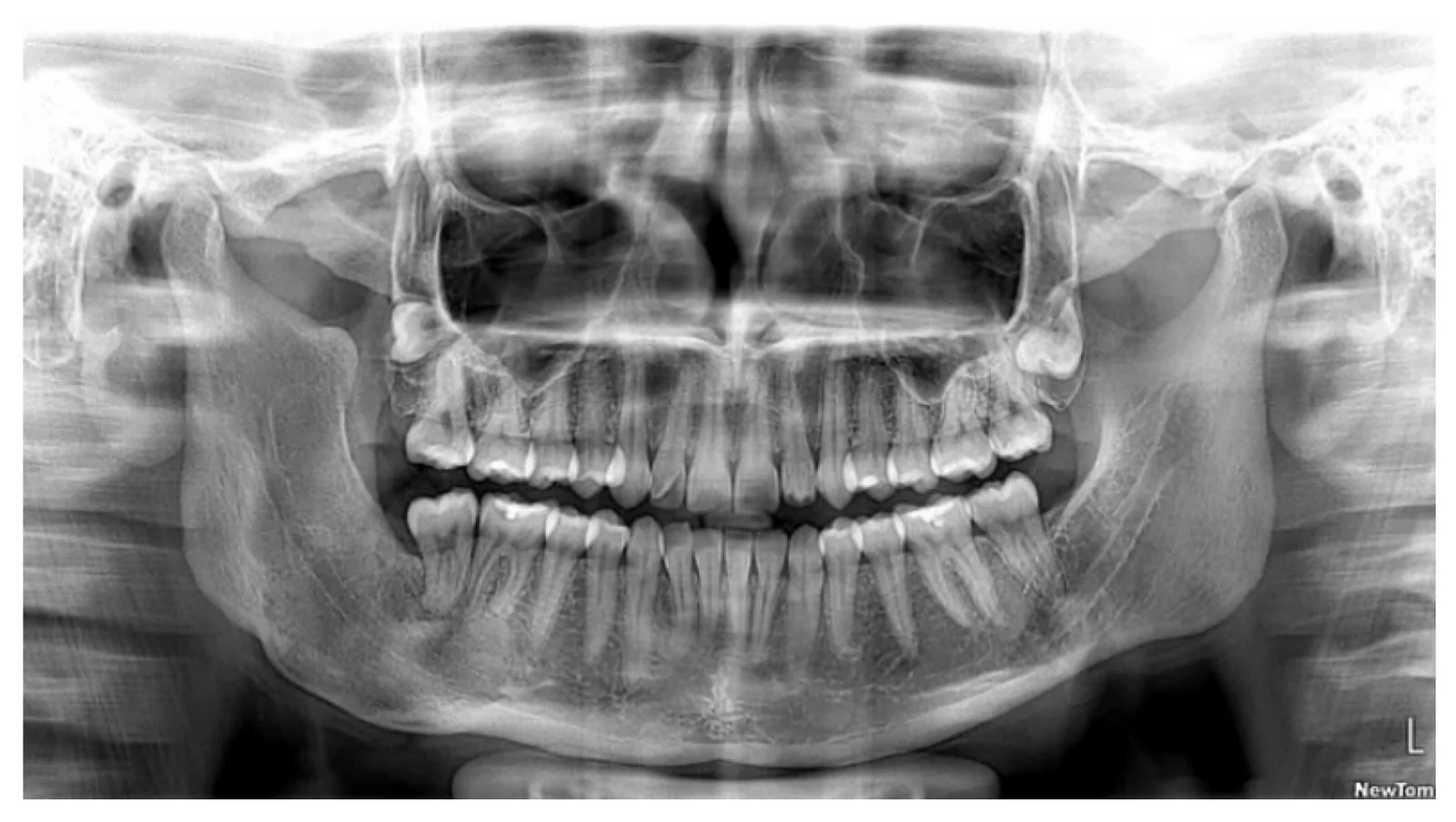
Panoramic radiograph at 2-year follow-up, showing maintained bone regeneration without evidence of recurrence or residual pathological tissue.

**Figure 9:**
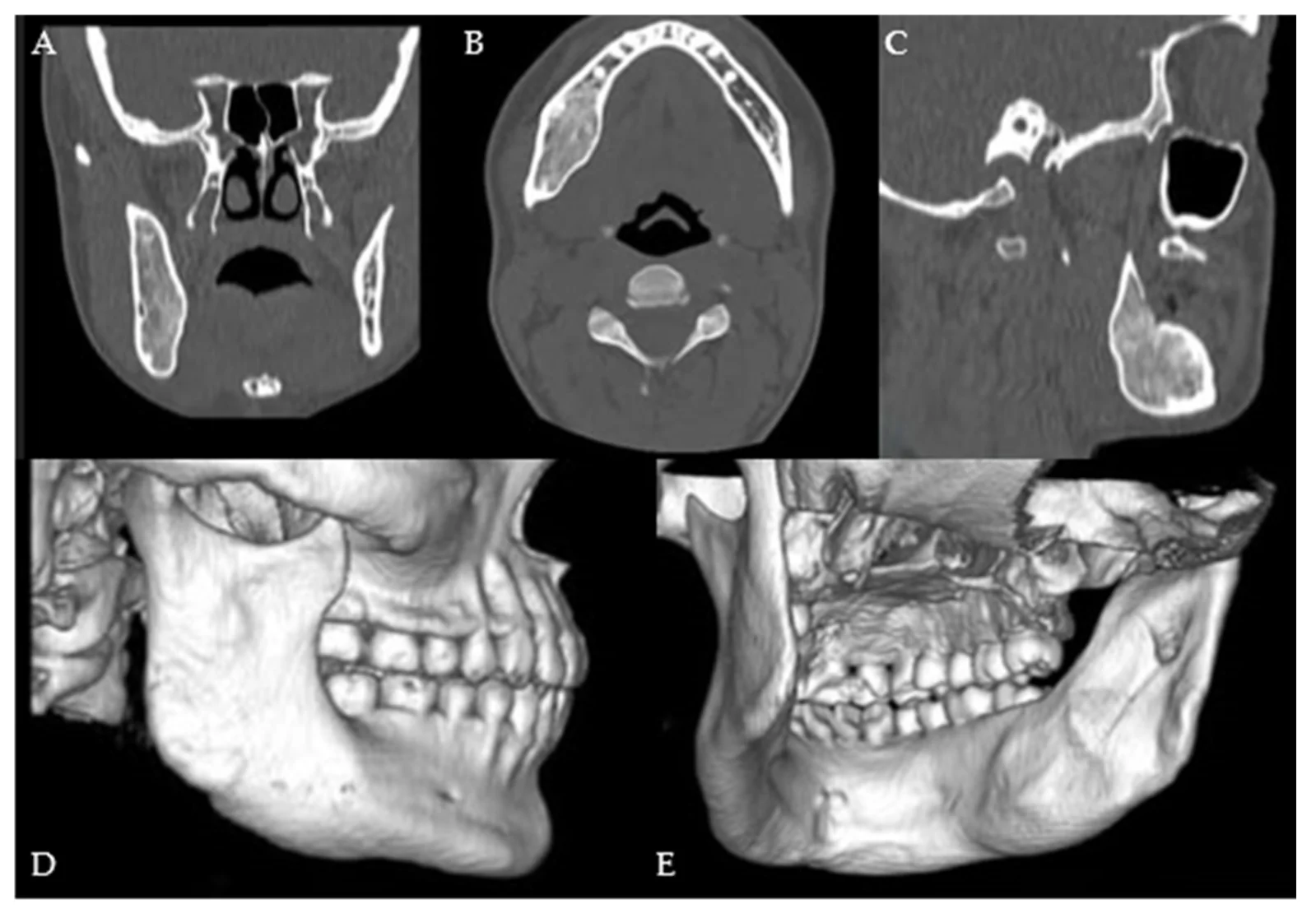
Cone-beam computed tomography (CBCT) at 2 years of follow-up. (**A**) Coronal, (**B**) axial, and (**C**) sagittal sections; (**D**) right lateral and (**E**) left posterior oblique views of the mandibular tomographic reconstruction, showing complete bone regeneration in the treated mandibular region without evidence of recurrence.

**Figure 10:**
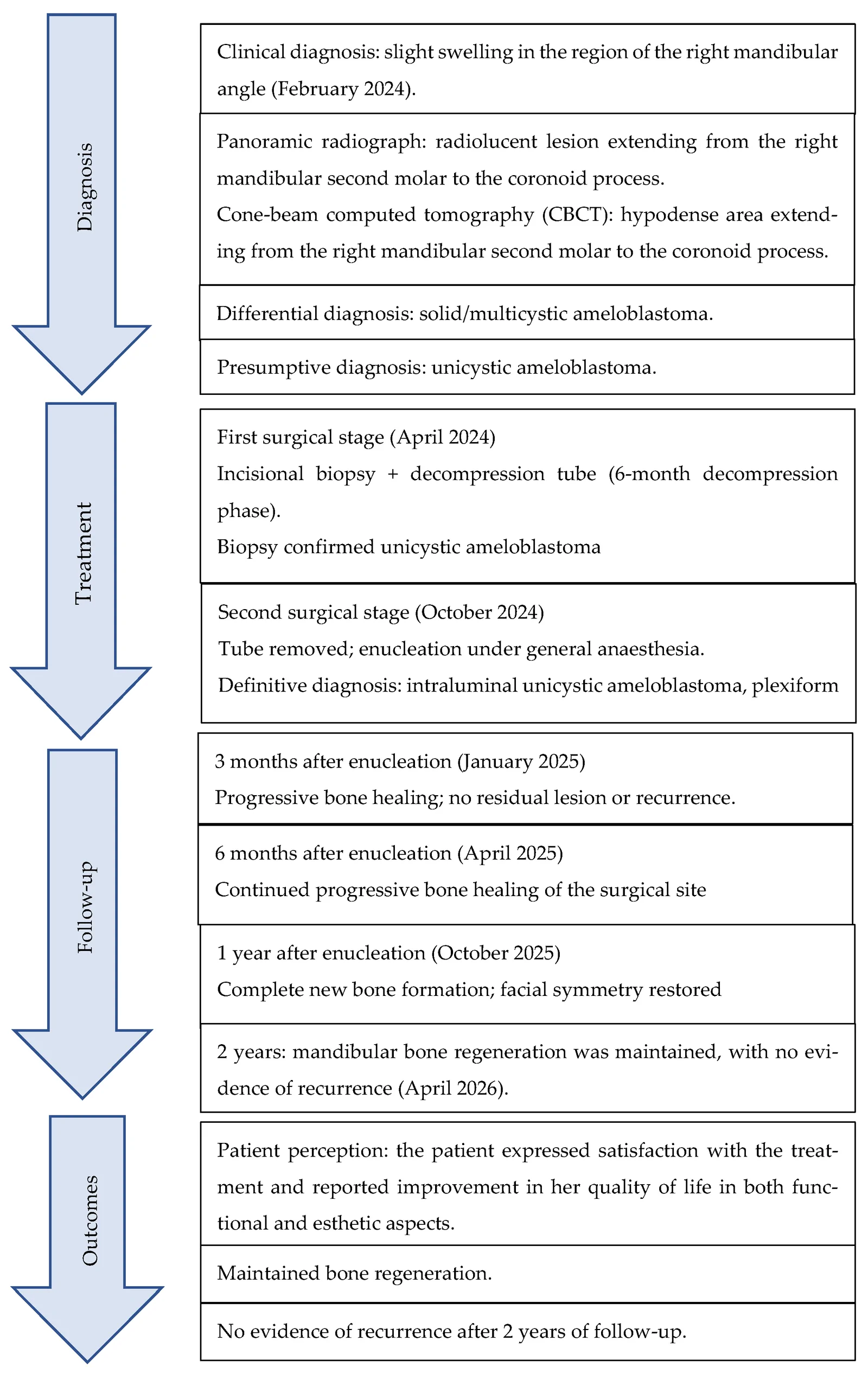
Flow diagram of the clinical case timeline.

At 2 years of follow-up after the initial surgical stage, the results of conservative treatment remained favorable. Follow-up panoramic radiography and CBCT (Figure 8 and Figure 9) showed no evidence of recurrence or residual pathological tissue, together with adequate bone regeneration in the treated mandibular region. The patient expressed satisfaction with the outcomes, reporting improvement in her quality of life in both functional and esthetic terms. The complete timeline of the case is presented in Figure 10.

## 3. Discussion

The present case documents conservative two-stage management of an intraluminal unicystic ameloblastoma through initial decompression followed by enucleation. Therapeutic planning was based on the criteria proposed by Gasparro et al. [6], who consider patient age, anatomical location, lesion dimensions, and histological subtype as key prognostic determinants.

McClary et al. [13] proposed that the intraluminal subtype, because it does not show invasion of the supporting connective tissue, represents the only histological variant suitable for conservative treatment, with a lower probability of recurrence. In agreement with this, Díaz-Reiher et al. [14] reported that decompression is indicated in the absence of imaging findings suggesting root resorption or cortical perforation, as these signs are associated with the mural subtype and greater biological aggressiveness. The present case fulfilled both criteria: a histopathologically confirmed intraluminal subtype and absence of cortical involvement or root resorption in the initial tomographic evaluation. The distinction between the intraluminal and mural subtypes rests exclusively on histomorphological examination of the connective tissue wall and therefore depends on adequate sampling of the entire capsule; this represented the principal diagnostic challenge in the present case and guided the histopathological protocol described above. In addition, no unerupted or impacted tooth was associated with the lesion, a finding that does not support a dentigerous origin and is consistent with a lesion arising de novo.

In cases showing imaging or histological features associated with the mural subtype and greater biological aggressiveness, chemical adjuncts such as Carnoy’s solution have been applied after enucleation to eliminate residual satellite tumor cells and reduce the risk of recurrence [4]. A retrospective series reported a recurrence rate of approximately 10% after enucleation combined with Carnoy’s solution, despite a high proportion of mural invasion, suggesting a possible protective effect [15,16]. However, a recent systematic review of Carnoy’s solution as an adjunct in jaw lesions found no strong evidence supporting its use in ameloblastoma and recommended prospective, randomized studies, as the available data derive from non-controlled series [16,17]. In the present case, the lesion was an intraluminal unicystic ameloblastoma without mural invasion, cortical perforation, or root resorption—features indicating lower biological aggressiveness—so decompression followed by enucleation was performed without chemical adjuncts; the role of such adjuncts appears more relevant for subtypes with mural invasion or higher recurrence risk.

Furthermore, Meenal et al. [18] proposed a therapeutic stratification according to the degree of invasion: ameloblastomas limited to the cystic lining or showing microinvasion of the connective wall are amenable to enucleation, whereas those with complete transmural invasion require segmental resection. This classification supports the decision to perform enucleation in the present case, given the intraluminal pattern without stromal invasion.

Ameloblastomas—particularly conventional (solid/multicystic) tumors with follicular or plexiform patterns—are known to have high recurrence rates after conservative treatment, which is an important consideration in the present case. However, in unicystic ameloblastoma, the plexiform architectural pattern is not, on its own, recognized as an independent determinant of recurrence risk; the granular-cell and, particularly, the desmoplastic subtypes are those most consistently associated with more aggressive behavior and a greater tendency to recur [19]. Here, biological behavior depends primarily on the extent of tumor proliferation—mural invasion being the histopathological finding with the greatest prognostic impact—rather than on the architectural pattern alone [20]. In the present case, the tumor was an intraluminal unicystic ameloblastoma without mural invasion, which is associated with a lower risk of recurrence than mural or conventional ameloblastomas despite the plexiform growth pattern.

Direct comparisons with the existing literature are limited, as few case reports specifically address conservative management of intraluminal unicystic ameloblastoma with a plexiform pattern [9,21]. Nevertheless, a two-stage approach comparable to ours has been reported in a mural unicystic ameloblastoma with a plexiform pattern treated by initial decompression followed by enucleation and curettage of the residual epithelium, with a favorable 2-year outcome and no need for mandibular resection; notably, that report still advocated strict clinical and radiographic surveillance over a 10-year period [14]. Similarly, Gaitán et al. [8] reported a unicystic ameloblastoma managed by 3 years of marsupialization followed by enucleation, with favorable results and no signs of recurrence at 4 years. These reports are consistent with our observation that conservative treatment can be effective for unicystic ameloblastoma, particularly in young patients (aged 20–30 years) with good adherence to follow-up and postoperative care.

Regarding prognosis, unicystic ameloblastoma of the intraluminal subtype—one of the least aggressive histological variants, with tumor proliferation confined to the cystic lumen and no invasion of the supporting connective tissue—is generally associated with a more favorable outcome and a lower likelihood of recurrence than the mural subtype or conventional ameloblastoma [22]. In the present case, the absence of mural invasion, cortical perforation, and root resorption, together with a favorable clinical and radiographic course at two years, supports a favorable prognosis, qualified by the recognized potential for late recurrence. Consequently, we suggest a prolonged follow-up protocol combining periodic clinical and radiographic (panoramic and, when indicated, CBCT) evaluation. Consistent with the surveillance advocated for comparable conservatively managed cases, we recommend maintaining regular monitoring over an extended period of up to 10 years, so that any late recurrence can be detected and managed promptly.

The risk–benefit balance between conservative and radical approaches is complex. Guo et al. [19] acknowledged that a radical approach minimizes recurrence but entails significant morbidity, including masticatory dysfunction, tooth loss, and facial deformity. In contrast, Lee et al. [4] noted that conservative treatment involves a higher risk of recurrence and requires active patient cooperation during the decompression phase. In the present case, the patient demonstrated adequate adherence to the follow-up protocol, which contributed to the favorable outcome. The possibility of recurrence carries direct clinical implications for this patient: should the lesion recur, a more aggressive approach—potentially segmental or marginal resection—might become necessary, with the associated morbidity of facial deformity, pathological fracture, tooth loss, neurosensory disturbance, and the need for complex reconstructive procedures for functional and esthetic rehabilitation. This prospect reinforces the importance of strict and prolonged clinical and radiographic follow-up to enable early detection and timely management of any recurrence.

One limitation of this case is the relatively short follow-up period compared with other studies. Unicystic ameloblastoma is recognized for its potential for late and unpredictable recurrence, and a 2-year follow-up from the initiation of treatment is therefore insufficient to establish a definitive cure, particularly as recurrence intervals of up to 7 years after treatment have been reported [23]. A further limitation is that no imaging was obtained during the decompression phase itself; therefore, the reduction in intracystic size attributable to decompression was not documented radiographically or quantified, and the intermediate radiographs available correspond to the postoperative period after enucleation. The effect of decompression in this case is thus supported by the clinical course and the final outcome rather than by serial imaging during that phase. Finally, no historical imaging predating the initial consultation was available. Consequently, it was not possible to establish whether the mandibular third molars were congenitally absent or had been lost previously, nor to reconstruct the earlier radiographic evolution of the lesion.

The strengths of this case include the involvement of a multidisciplinary team, an accurate histopathological diagnosis that allowed the selection of the most appropriate treatment, and adequate follow-up documented clinically and through imaging studies. Patient cooperation was also essential, and favorable results were achieved after decompression and enucleation of the lesion, with apposition of the mandibular bone and no recurrence after 2 years of follow-up from the initiation of treatment.

## 4. Conclusions

An accurate and timely clinical, radiographic, and histopathological diagnosis is essential to plan the definitive surgical treatment of unicystic ameloblastoma. In the present case, conservative two-stage management—decompression followed by enucleation—achieved a favorable outcome, with mandibular bone apposition and no evidence of recurrence at two years from the initiation of treatment. Nevertheless, each case must be individualized, and long-term clinical and radiographic surveillance remains necessary to rule out late recurrence before conservative treatment can be considered definitively successful.

## Data Availability

The data presented in this study are available on request from the corresponding author due to privacy restrictions.

## References

[1] World Health Organization (2022). WHO Classification of Head and Neck Tumours.

[2] Anpalagan A., Tzortzis A., Twigg J., Wotherspoon R., Chengot P., Kanatas A. (2021). Current practice in the management of peripheral ameloblastoma: A structured review. Br. J. Oral Maxillofac. Surg..

[3] Rayamajhi S., Shrestha S., Shakya S., Bhandari S., Twayana A.R., Shahi K. (2022). Unicystic Ameloblastoma of Mandible: A Case Report. J. Nepal Med. Assoc..

[4] Lee S.M., Ku J.K., Leem D.H., Baek J.A., Ko S.O. (2021). Conservative management with Carnoy’s solution in ameloblastoma involving two unerupted teeth: A report of two cases. J. Korean Assoc. Oral Maxillofac. Surg..

[5] Chaudhary Z., Sharma P., Hemavathy S., Joshna E.K., Augustine J., Vijayaragavan R., Nehra A., Garg V. (2024). Unicystic ameloblastoma: Clinico-radiological and histopathological correlation with management. J. Plast. Reconstr. Aesthetic Surg..

[6] Gasparro R., Giordano F., Campana M.D., Aliberti A., Landolfo E., Dolce P., Sammartino G., di Lauro A.E. (2024). The Effect of Conservative vs. Radical Treatment of Ameloblastoma on Recurrence Rate and Quality of Life: An Umbrella Review. J. Clin. Med..

[7] Hresko A., Burtyn O., Pavlovskiy L., Snisarevskyi P., Lapshyna J., Chepurnyi Y., Kopchak A., Karagozoglu K.H., Forouzanfar T. (2021). Controversies in ameloblastoma management: Evaluation of decision making, based on a retrospective analysis. Med. Oral Patol. Oral Cir. Bucal.

[8] Gaitán Sequeira R., Arrascue Dulanto M., Hidalgo Chávez J. (2025). Tratamiento Conservador de un Ameloblastoma uniquístico: Reporte de caso con cuatro años de seguimiento. Rev. Odontológica Stomarium.

[9] Zheng C.Y., Cao R., Hong W.S., Sheng M.C., Hu Y.J. (2019). Marsupialisation for the treatment of unicystic ameloblastoma of the mandible: A long-term follow up of 116 cases. Br. J. Oral Maxillofac. Surg..

[10] Rocha A.C., Fonseca F.P., Santos-Silva A.R., Lourenço S.V., Ceccheti M.M., Júnior J.G. (2021). Effectiveness of the Conservative Surgical Management of the Ameloblastomas: A Cross-Sectional Study. Front. Oral Health.

[11] Demir E., Gunhan O. (2023). Conservative treatment of a unicystic ameloblastoma by marsupialization with a favorable response: A case report and review of the literature. Dent. Res. J..

[12] Sohrabi C., Mathew G., Maria N., Kerwan A., Franchi T., Agha R.A. (2023). The SCARE 2023 guideline: Updating consensus Surgical CAse REport (SCARE) guidelines. Int. J. Surg..

[13] McClary A.C., West R.B., McClary A.C., Pollack J.R., Fischbein N.J., Holsinger C.F., Sunwoo J., Colevas A.D., Sirjani D. (2016). Ameloblastoma: A clinical review and trends in management. Eur. Arch. Oto-Rhino-Laryngol..

[14] Díaz-Reiher M., Yévenes-Souper F., Fernández-Toro M.d.l.Á., Donoso-Hofer F. (2022). Ameloblastoma uniquístico patrón mural: Reporte de un caso. Int. J. Interdiscip. Dent..

[15] Lee P.K., Samman N., Ng I.O. (2004). Unicystic ameloblastoma—Use of Carnoy’s solution after enucleation. Int. J. Oral Maxillofac. Surg..

[16] Qiao X., Shi J., Liu J., Liu J., Guo Y., Zhong M. (2021). Recurrence Rates of Intraosseous Ameloblastoma Cases With Conservative or Aggressive Treatment: A Systematic Review and Meta-Analysis. Front. Oncol..

[17] Lal B., Kumar R.D., Alagarsamy R., Shanmuga Sundaram D., Bhutia O., Roychoudhury A. (2021). Role of Carnoy’s solution as treatment adjunct in jaw lesions other than odontogenic keratocyst: A systematic review. Br. J. Oral Maxillofac. Surg..

[18] Meenal V., Nikhil V., Madhusudan A. (2023). Conservative Management of Unicystic Ameloblastoma of Mandible Evolving from Dentigerous Cyst in a Paediatric Patient: A Case Report. J. Dent..

[19] Guo T., Zhang C., Zhou J. (2022). Unicystic ameloblastoma in a 9-year-old child treated with a combination of conservative surgery and orthodontic treatment: A case report. Clin. Case Rep..

[20] Heikinheimo K., Huhtala J.M., Thiel A., Kurppa K.J., Heikinheimo H., Kovac M., Kragelund C., Warfvinge G., Dawson H., Elenius K. (2019). The Mutational Profile of Unicystic Ameloblastoma. J. Dent. Res..

[21] Liceaga-Escalera C., Montoya-Pérez L.A., Vélez-Cruz M., Jiménez-de la Puente G. (2020). Ameloblastoma uniquístico tratado mediante descompresión y enucleación. Reporte de un caso y revisión de la literatura. Rev. Odontol. Mex..

[22] Gültekin S.E., Büttner R. (2022). Clinical and pathomorphological aspects of odontogenic tumors. Die Pathol..

[23] Vinitzky-Brener I., Harfuch-Capdevila T., Abascal-Quintana C., Aldape-Barrios B. (2022). Tratamiento conservador de ameloblastoma uniquístico. Presentación de un caso. Odontol. Sanmarquina.

